# The anterior insula bidirectionally modulates cost‐benefit decision‐making on a rodent gambling task

**DOI:** 10.1111/ejn.13689

**Published:** 2017-10-06

**Authors:** M. L. Daniel, P. J. Cocker, J. Lacoste, A. C. Mar, J. L. Houeto, A. Belin‐Rauscent, D. Belin

**Affiliations:** ^1^ Department of Psychology University of Cambridge Downing Street Cambridge CB2 3EB UK; ^2^ Service de Psychiatrie et Addictologie CHU de Martinique Fort de France Cedex France; ^3^ Department of Neuroscience and Physiology Neuroscience Institute New York University School of Medicine New York NY USA; ^4^ Service de Neurologie CIC‐INSERM 1402 CHU de Poitiers Poitiers Cedex France

**Keywords:** decision‐making, gambling task, individual differences, insular cortex

## Abstract

Deficits in cost‐benefit decision‐making, as assessed in the Iowa Gambling Task (IGT), are commonly observed in neuropsychiatric disorders such as addiction. There is considerable variation in the maximization of rewards on such tasks, both in the general population and in rodent models, suggesting individual differences in decision‐making may represent a key endophenotype for vulnerability to neuropsychiatric disorders. Increasing evidence suggests that the insular cortex, which is involved in interoception and emotional processes in humans, may be a key neural locus in the control of decision‐making processes. However, the extent to which the insula contributes to individual differences in cost‐benefit decision‐making remains unknown. Using male Sprague Dawley rats, we first assessed individual differences in the performance over the course of a single session on a rodent analogue of the IGT (rGT). Rats were matched for their ability to maximize reward and received bilateral excitotoxic or sham lesions of the anterior insula cortex (AIC). Animals were subsequently challenged on a second rGT session with altered contingencies. Finally, animals were also assessed for instrumental conditioning and reversal learning. AIC lesions produced bidirectional alterations on rGT performance; rats that had performed optimally prior to surgery subsequently showed impairments, and animals that had performed poorly showed improvements in comparison with sham‐operated controls. These bidirectional effects were not attributable to alterations in behavioural flexibility or in motivation. These data suggest that the recruitment of the AIC during decision‐making may be state‐dependent and help guide response selection towards subjectively favourable options.

## Introduction

Deficits in cost‐benefit decision‐making are commonly observed in neuropsychiatric disorders such as addiction (Bechara *et al*., 2001; Cavedini *et al*., 2002). The Iowa Gambling Task (IGT) is one of the most frequently used laboratory measures designed to simulate ‘real‐life’ decision‐making under uncertainty (Bechara *et al*., 1994). During the IGT, subjects are instructed to choose cards from one of four decks to accumulate points. Two decks offer higher immediate gains but also confer occasional large losses and are disadvantageous in the long term. The other two decks yield smaller gains and losses and choosing them is advantageous towards an overall gain in points. The contingencies are unknown to the subjects but the majority learn to select from the two optimal decks over the course of a single session. However, there is considerable variability between participants in their ability to maximize reward on this task, with roughly a third of healthy subjects continuing to choose primarily from the disadvantageous decks (Bechara *et al*., 1994; Bechara & Damasio, 2002; Steingroever *et al*., 2013). Increased choice of the risky decks has also been reported in subjects with behavioural and substance addictions (Grant *et al*., 2000; Cavedini *et al*., 2002) as well as in patients with damage to prefrontal regions, putatively due to a deficit in bioregulatory feedback that guides optimal decision‐making in this task (Bechara *et al*., 1994, 1997; but see Maia & McClelland, 2004; Bechara *et al*., 2005).

The insular cortex has been proposed to integrate both environmental and somatic information in order to generate cohesive interoceptive representations, indicating that it may be importantly engaged in IGT performance (Craig, 2002). Human subjects show increased activity in the insula when performing the IGT (Tanabe *et al*., 2007; Krawitz *et al*., 2010; Li *et al*., 2010). However, as the insula is actively engaged in monitoring multiple aspects of behaviour, changes in activity during IGT performance may reflect epiphenomenon as a result of alterations in autonomic arousal, ergo a definitive contribution of the insula to IGT performance in humans is unclear (see Sterzer & Kleinschmidt, 2010 for review).

The human insula can be roughly divided into agranular and granular regions. Based on connectivity and cytoarchitectonics, these regions are broadly analogous to the rodent anterior insular cortex (AIC) and posterior insular cortex, respectively (Allen *et al*., 1991; Singer *et al*., 2009). The AIC is the predominant output domain of the insula sending projections to key components of the amygdalo‐frontostriatal circuitry. It is engaged in modulating impulsivity, compulsivity, associative learning and responsivity to reward associated stimuli (Reynolds & Zahm, 2005; Li *et al*., 2013; Belin‐Rauscent *et al*., 2016; Cocker *et al*., 2016). The AIC has also recently been shown to bidirectionally influence the development and expression of escalation of cocaine self‐administration in rats allowed extended access to the drug (Rotge *et al*., 2017). Thus, the AIC is an intriguing candidate area that might contribute importantly to individual differences in cost‐benefit decision‐making.

Previous studies utilizing different rodent versions of the IGT have further suggested an involvement of the insula in cost‐benefit decision‐making, although the results are equivocal. AIC lesions and inactivations increased choice of smaller more certain rewards in one study (Pushparaj *et al*., 2015b), whereas chemogenetic inhibition of the insula increased choice of a high‐risk high reward option, using a maze based task (Mizoguchi *et al*., 2015). However, both of these paradigms utilized relatively extensive training regimes and did not address the potential contribution of the AIC to inter‐individual differences in decision‐making. Moreover, as the AIC has been suggested to modulate bioregulatory feedback used to guide behaviour, perhaps the most critical period to examine would be across a single session, wherein animals have to initially learn about the relative contingencies of different options, that is rely on an exploratory strategy, before using this knowledge to maximize reward during the latter part of the session, a strategy referred to as exploitation (Bechara *et al*., 1997; Craig, 2002, 2009b).

To causally determine the role of the AIC in inter‐individual differences in decision‐making, we measured the impact of bilateral excitotoxic lesions of the AIC on individual performance in an operationalized version of the IGT – the rat gambling task (rGT). The rGT has the advantage of capturing inter‐individual differences in decision‐making across the course of a single session. In the rGT, rats first sample from four different options to learn about their relative utility without prior knowledge. Over trials, rats must learn to avoid the superficially alluring but ultimately disadvantageous options in order to maximize rewards (food) and minimize punishments (timeout periods) within a time constrained session (Rivalan *et al*., 2009). Such maximization of reward has been recently shown to be associated with the functional recruitment of a broad cortico‐striatal system that includes the insula (Fitoussi *et al*., 2015), as measured by c‐fos expression in rats performing the rGT.

## Materials and methods

### Subjects

**Figure 1:**
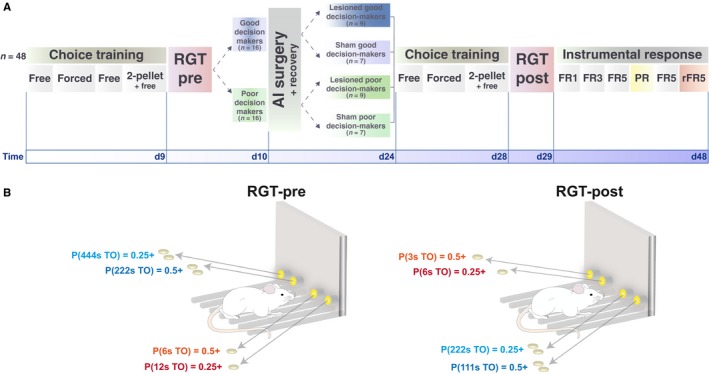
General experimental design and rGT choice structure. (A) The behavioural procedure before surgery consisted of nine daily sessions (see details in Materials and methods). Animals initially responded in any of the four active holes during free choice sessions to obtain one food pellet. Forced choice sessions subsequently ensured that animals explored every active hole. Two further free choice sessions were then administered, during which animals received two‐sugar pellets for half of each session. Rats were then tested on a single pre‐surgery rGT session from which good (*n* = 16) and poor (*n* = 16) decision‐makers were identified according to their performance. Rats underwent either bilateral AI‐ or sham lesion (respectively, *n* = 9 and *n* = 7). After recovery, animals were exposed to similar training sessions, but over 4 days and finally a post‐surgery rGT session was conducted. The second rGT was based on the same principle of disadvantageous and advantageous two‐hole options but utilised a new combination of conditions to avoid learning effects. To further explore the effects of AIC lesions, the acquisition of instrumental responding for food reward in a two‐lever operant conditioning chamber under increased FR response requirements (FR1, FR3 and FR5) was measured. Subsequently a PR schedule during which the cost of the reward is progressively increased within a single session measured animals’ motivation for food reward. Lastly, rats’ ability to update or alter their behaviour was measured via five reversal learning sessions according to a FR5 schedule under reversed values of the levers (FR5r). (B) Diagram showing the utility of the four options in each of the two rGT sessions. Rats are offered four options, two of which were associated with small reward (one pellet) and the other two with higher reward (two pellets, represented here pictorially). The higher rewards were associated with higher punishment in the form of time–out periods, the duration of which is shown in seconds along with the relative probability (*P*). Timeouts were delivered concomitant with reward. The options delivering single pellets were considered advantageous due to the lower potential timeout punishments. Thus, rats had to sample the various options and learn to move away from high reward/high cumulative loss choices to low reward/low cumulative loss choices. Advantageous options were counterbalanced against the animals preferred side during training. During the second rGT, the contingencies were reshuffled and the duration of penalties was altered so animals had to sample each hole again in order to re‐learn the optimal strategy. [Colour figure can be viewed at wileyonlinelibrary.com].

Male Sprague Dawley rats (*n* = 48; Charles River, Arbresle, France) weighing ~250 g at arrival were pair‐housed in a climate controlled environment, temperature: 22 ± 1 °C, humidity: 60 ± 5% under a reversed 12‐h light/dark cycle (light on at 7:00 PM). Animals were fed standard rat chow and 1 week before the start of testing were food restricted to ~85–90% of their free feeding weight. Water was available *ad libitum*. Experiments were conducted between 1:00 PM and 7:00 PM 5–7 days per week. This research has been regulated under the Animals (Scientific Procedures) Act 1986 Amendment Regulations 2012 following ethical review by the University of Cambridge Animal Welfare and Ethical Review Body (AWERB). A timeline of the experiment is presented in Fig. 1A.

### Surgery

Animals were anaesthetized using intraperitoneal injection of avertin (10 mL/kg) and received either AIC lesions (*n* = 24) or sham surgery (*n* = 24) using standard stereotaxic techniques. Bilateral infusions of 0.8 μL of 0.09 m quinolinic acid or vehicle were delivered via a 26‐gauge cannula (Phymep, Paris, France) connected to a 10‐μL Hamilton syringe at the following coordinates: anterior/posterior + 1.44 mm and medial/lateral ± 5.2 mm from bregma, dorsal/ventral – 6.8 mm from skull (Paxinos and Watson, 1986), incisor bar at 3.3 mm. Each infusion was made over 2 min 30 s and allowed to diffuse for an additional 2 min prior to removal of the injector. Rats were allowed to recover for 8 days before testing resumed.

### The rat Gambling Task

Testing took place in six standard five‐hole operant chambers (Med. Associates, St Albans, VT, USA). Each chamber was located within a sound attenuating chamber equipped with exhaust fans that assured air renewal and masked background noise. Operant chambers were illuminated by a white 3‐W house light during experimental sessions. A pellet dispenser delivered 45‐mg dustless precision pellets (Bio‐Serv Inc., NJ, USA) to a food magazine on the right wall. On the opposite wall the five‐hole stimulus array was positioned 2 cm above a bar floor and each aperture contained a stimulus light. Nose pokes into the food magazine or the holes were recorded with an infrared photobeam. The boxes were controlled by software written in med‐pc (Med. Associates) on a computer running under Windows 7.

Animals were exposed to sucrose pellets in their home cages before being habituated to the testing boxes. During initial magazine training, 60 pellets were delivered to the magazine on a 30‐s variable interval schedule. A schematic of the training and testing timeline is provided in Fig. 1. Initially rats were trained to nose poke into one of the four lateral illuminated holes to receive a food‐pellet reward. Responses in the middle, inoperative hole were recorded but had no programmed consequence. Sessions continued until rats obtained 100 pellets or 30 min elapsed. After two free choice training sessions, rats were given four forced choice 30‐min sessions during which, one of the four holes was active for 7 min 30 s on a pseudorandom schedule. Forced choice sessions were implemented to help animals avoid development of a side or hole bias. Subsequently, animals underwent two free choice sessions. In the second of these each nose poke in any of the four active holes provided two pellets during the first half of the session and one pellet during the second half. During these last free choice sessions side preferences were characterized for each rat.

During the rGT proper, two of the four active holes, defined as the disadvantageous holes, resulted in the guaranteed delivery of two pellets but a potential long timeout of either 222 or 444 s with a probability of 0.5 and 0.25, respectively. The other two active holes, defined as the advantageous holes, were associated with the delivery of only one pellet but potentially followed by a short timeout of either 6 or 12 s with respective probabilities of 0.5 and 0.25. The probability of receiving a timeout punishment for each hole was fixed for the duration of the session. Both advantageous options were located on one side of the chamber, but the side of the advantageous holes was counterbalanced with the side of any potential preference previously identified. The test session lasted until rats obtained 250 pellets or 60 min had elapsed. A configuration was assigned to each rat: the side of the advantageous holes was counterbalanced with its side preference previously identified.

As the utility within each pair of options was similar, choice of both advantageous option was pooled, as were choices from either disadvantageous option, to generate a decision‐making score for each animal, consistent with Rivalan *et al*. (2013). The ratio of advantageous choices during the first 15 first minutes of the test, the so‐called exploration phase, was compared to advantageous choices during the last 15 min of the test when animals were theoretically maximizing rewards given current information, the so‐called exploitation phase. The top third of the cohort had scores greater than 20 and were categorized as good decision‐makers (GDM, *n* = 16). Animals in the bottom third had scores below or equal to 2.5 and were labelled as poor decision‐makers (PDM, *n* = 16). All animals with intermediate scores were discarded form further analysis. Animals were matched for task performance and underwent surgery and recovery as previously described.

Following recovery, animals were re‐baselined with four training sessions to determine any existing side preference: one free choice session, two forced choice sessions and finally another free choice session during which nose pokes resulted in the delivery of two pellets for the first part and one pellet for the second part of the session. Animals then underwent a second rGT session, during which contingencies were reshuffled and variations in the duration of penalties were systematically introduced to prevent a learning effect due to the first testing. As illustrated in Fig. 1B, poking in the disadvantageous holes resulted in the delivery of two pellets with respective probabilities of 0.25 and 0.5 to be followed by a long timeout of 222 and 111 s, respectively, whereas the advantageous holes were associated with the delivery of only one pellet with respective probabilities of 0.25 and 0.5 to be followed by a short timeout of 6 and 3 s, respectively. Advantageous holes during rGT were also counterbalanced with side preference defined on the basis of previous free choice performance to avoid potential side bias.

### Instrumental conditioning

In order to ensure that any observed behavioural effects following AIC lesions were not due to alterations in motivation or behavioural flexibility, animals were trained on a simple instrumental conditioning and reversal learning paradigm. To avoid any confounding effects of prior rGT performance different operant boxes were used. These were 12 identical chambers (30 × 24 × 21 cm, similar to the ones used for the rGT, but without the five‐hole hole wall and with the addition of two retractable levers, with cue lights above, located either side of a food delivery magazine. Experimental contingencies were controlled and data collected with med‐pc software (Med Associates).

During training and all subsequent testing one lever was assigned as the active lever and the other as the inactive lever. Responses on the inactive lever were recorded but had no programmed consequence. During the first session lever responses on the active lever were rewarded on a fixed‐ratio 1 (FR1) schedule. FR1 sessions were followed by FR3 and FR5 schedules of reinforcement. All FR sessions lasted for 30 min or until 100 pellets were delivered.

Animals’ motivation to obtain reward was tested using a single session of progressive ratio (PR). The response requirement was 5, 9, 12, 15, 20, 25, 32, 40, 50, 62, 77, 95, 118, 145, 178, 219, 268, 328, 402, 492, 603, 737, 901, 1102, 1347, 1647 and 2012. The session ended after 90 min. The final response requirement met was defined as the breakpoint.

After two additional FR5 sessions, behavioural flexibility was assessed by switching the lever contingencies such that the previously inactive lever was now active and the active was inactive. Animals had five daily sessions to examine how quickly animals extinguished former associations and acquired the new contingencies.

### Histology

Following completion of behavioural testing, rats were deeply anesthetized with intraperitoneal administration of ketamine (Ketalar^®^ 100 mg/kg, Panpharma, France) and xylazine (Rompun^®^ 1 mg/kg, Bayer, Puteaux, France) and perfused transcardially with 0.1 m phosphate buffer (pH = 7.4), followed by 4% paraformaldehyde in 0.1 m phosphate buffer. Brains were then removed and placed in 4% formaldehyde before being transferred to a 30% sucrose solution for at least 24 h before being sectioned at 40 μm using a freezing microtome. Sections were mounted and stained with Cresyl Violet. Slides were examined for placement and extent of the lesion.

### Statistical analyses

All statistical analyses were conducted using spss (version 24) and statistica 10 (Statsoft). Animals were classified as GDM and PDM on the basis of their ability to learn the optimal strategy during the first rGT, parameterized as the ratio of good to bad choices in the exploration phase, in comparison with the exploitation phase in a similar manner to Rivalan *et al*. (2013). The percentage of advantageous choices during the first 15 min and the last 15 min of each individual session was calculated. This calculation was used as it quantified animals’ shifts in preference over a time‐locked schedule and therefore most accurately reflected learning. However, as the task incorporates timeouts as punishments, the number of trials the animals can complete within these time frames is variable. Therefore, choices were grouped into four blocks across the session (0–25, 25–50, 50–75, 75–100% of trials completed) for statistical analysis. We were thus able to compare choice across the session between animals independent of the number of timeouts or of the associated number of completed trials. Data were subjected to an arcsine transformation to limit the impact of an artificial ceiling (i.e. 100%; McDonald, 2009). Sphericity was assessed using Mauchly's test and, when violated, was corrected for using Greenhouse–Geisser.

Advantageous choices in the first and second rGT were analysed separately as repeated‐measures anovas with trial block (four levels) as a within‐subjects factor, and decision‐making classification (two levels) and surgery group (two levels) as between‐subjects factors. Data from instrumental conditioning were analysed using similarly structured anova except that the trial block factor was lever (active/inactive; two levels). Where appropriate, *post hoc* comparisons were made using Newman–Keuls test. The significance level for all effects was *P* ≤ 0.05. Analyses for which *P* ≤ 0.1 were described as trends. Effect sizes are reported using partial η^2^ (*p*η^2^). All data are presented as mean ± SEM.

## Results

Three animals (two in the lesioned group and one in the sham group) died during surgery and were excluded from the analyses. No animals were excluded based on lesion placement.

**Figure 2:**
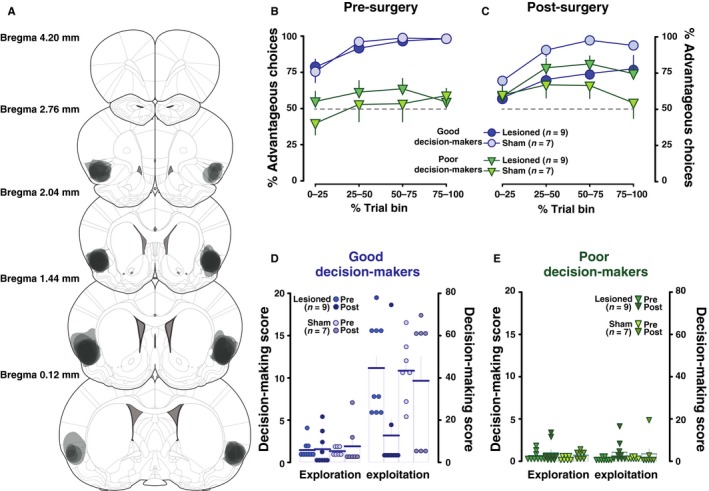
Bilateral AIC lesions impair maximization of reward in rats performing optimally in a rat gambling task. (A) Schematic representation of the extent of the bilateral excitotoxic lesions of the AIC from rats identified as good (*n *=* *16) and poor (*n *=* *16) decision‐makers. Areas shaded in grey represent the extent of neuronal damage. Coronal sections are 4.20 mm anterior through 0.12 mm anterior to the bregma. (B and C) Behavioural performance in the rGT of GDM and PDM rats was not similarly affected by AI lesion. Thus, sham GDM rats, akin to sham PDM, displayed similar performance in rGT prior to, and after surgery. In contrast, GDM lesioned animals showed an impairment in rGT performance as compared to their pre‐lesion test, and PDM lesioned animals exhibited an improvement in their performance, so that the performances of these two groups are similar across the course of the second rGT session. (D) During the exploration phase of the rGT, all good decision‐maker (GDM) rats tended to randomly explore all available options; the decision‐making score defined by the ratio of advantageous choices relative to disadvantageous choices for the same period was around 1 in both lesioned (*n *=* *9) and sham (*n *=* *7) groups before and after surgery. During the last minutes of the test, in this subpopulation the aim of maximizing rewards defined an exploitation phase; it was reflected by a marked preference for advantageous choices. There were around 40 advantageous choices for one disadvantageous choice in lesioned rats before surgery, and in sham rats before and after surgery. But on the other hand, good decision‐makers after AIC lesion showed a ~70% decrease in their decision‐making score. (E) In poor decision‐makers, the exploration phase was also similar through the different groups before and after surgery. Before surgery, in the last minutes of the test, when exploitation of gathered information about options would have been possible, both groups failed to identify the advantageous options as preferable and pursued risky choices; however, lesioned (*n *=* *9) and sham (*n *=* *7) PDM rats increased their exploitation performance by, respectively, 5.5 and 2.5 times during the second rGT despite recombined but matching conditions as compared to the previous one. Data are group means and SEM. Each dot represents an individual. [Colour figure can be viewed at wileyonlinelibrary.com].

The rGT requires animals to learn over the course of a single session to avoid the options associated with larger, immediate reward but with high risk of punishment, and instead to select from the two options associated with lower instant rewards, but also lower cumulative punishments. All animals showed a shift in choice selection throughout a single session, broadly increasing their choice of the advantageous options (Fig. 2B; main effect of trial block: *F*_3,84_ = 27.71, *P* < 0.0001, *p*η^2^ = 0.5). This effect was, as expected based on the grouping criteria, far more pronounced in GDM, who demonstrated progressive choice bias towards the advantageous options (main effect of group: *F*_1,28_ = 67.1, *P* < 0.0001, *p*η^2^ = 0.71 and trial block × group interaction: *F*_3,84_ = 8.81, *P* < 0.0001, *p*η^2^ = 0.24). Animals within GDM and PDM groups were matched for rGT performance and underwent bilateral lesion of the AIC (Fig. 1A), and there were no statistical differences between the two surgery groups (surgery group: *F*_1,28_ = 0.23, *P* = 0.64).

AIC lesions (Fig. 2A) produced state‐dependent alterations in maximization of reward across the course of a new rGT session in that AIC lesions abolished initial differences between GDM and PDM rats, related to an impairment in task performance in GDM and an improvement in PDM (main effect of surgery: *F*_1,28_ = 0.42, *P* = 0.52, surgery × group: *F*_1,28_ = 4.3, *P* = 0.05, *p*η^2^ = 0.13; trial block × surgery × group: *F*_3,84_ = 3.13, *P* = 0.04, *p*η^2^ = 0.1; Fig. 2B–E). As predicted by this observation, similar bilateral AIC lesions had no influence over performance of rats of the heterogeneous intermediate group, that displayed average levels of performance (data not shown; main effect of surgery: *F*_1,11_ = 0.14, *P* = 0.71 and surgery × block interaction: *F*_3,33_ = 0.94, *P* = 0.43).

The pronounced difference observed prior to surgery between GDM and PDM rats was reduced to a weak trend after the lesion (main effect of group: *F*_1,28_ = 3.3, *P* = 0.08, *p*η^2^ = 0.11), thereby further demonstrating that AIC lesions differentially influenced the performance of rats based on their ability to establish the optimal strategy in the task. Examining these effects further revealed that whilst there were still significant differences between GDM and PDM in sham‐operated animals (main effect of group: *F*_1,12_ = 10.21, *P* = 0.008, *p*η^2^ = 0.46; Fig. 2C) these differences were absent in lesioned animals (*F*_1,16_ = 0.03, *P* = 0.87).

The differential effect of AIC lesions on choice behaviour was even more pronounced when comparing the individuals’ use of exploration and exploitation strategies (main phase × group interaction: *F*_1,28_ = 123.19, *P* < 0.001, *p*η^2^ = 0.82; Fig. 2D–E). Thus, during the first rGT rats classified as GDM initially adopted an exploration strategy, sampling from the four options, before adopting a more efficient exploitation strategy, selecting almost exclusively from the advantageous options (main effect of phase: *F*_1,14_ = 215.34, *P* < 0.001, *p*η^2^ = 0.94). In contrast PDM rats failed to show a transition towards exploitation during the last 15 min of the session and instead persisted in an exploratory‐like pattern of choice (main effect of phase: *F*_1,14_ = 0.003, *P* = 0.96; Fig. 2D). However, these group differences were abolished in the second rGT (main effect of phase × group interaction: *F*_1,28_ = 0.02, *P* = 0.89 and phase × group × surgery interaction: *F*_1,28_ = 4.72, *P* = 0.02, *p*η^2^ = 0.14). This was attributable to a marked drop in performance in the exploitation phase of the GDM rats. *Post hoc* analyses confirmed that sham GDM rats still differed from all PDM rats during the exploitation phase of the second rGT (all *P*‐values < 0.001), but lesioned GDM no longer differed from lesioned and sham poor decision‐makers (all *P*‐values > 0.05). The increase in advantageous choice of lesioned PDM during the exploitation phase failed to reach statistical significance (all *P*‐values > 0.05; Fig. 2E).

**Figure 3:**
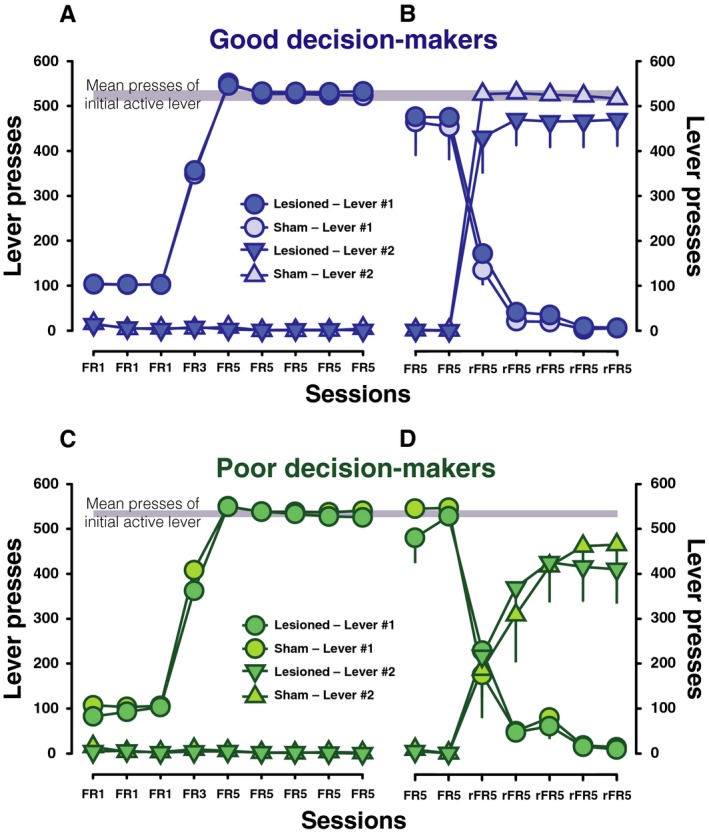
Preserved acquisition of instrumental response in all rats and impaired behavioural flexibility only in PDM. (A and C) Neither decision‐making trait nor AIC lesion affected the acquisition of instrumental conditioning. The total number of lever #1 presses, *that is* current active lever presses, increased in parallel with the increased behavioural requirement from FR1 to FR5 in both lesioned and sham operated good and poor decision‐makers. Lever #2 presses, *that is* current inactive lever presses, remained low in all groups. (B) After two additional FR5 sessions in which only lever #1 is active, the contingencies were reversed for five sessions such that lever #2 is now active (rFR5). In both lesioned and sham good decision‐makers, after switching, the total number of lever #1 presses decreased in parallel with the increased total number of lever #2 presses. Active lever presses summed for the five FR5 sessions of the acquisition phase and for the five rFR5 sessions of the reversal phase did not differ in these groups. (D) PDM under the same reversal conditions decreased their responses on the now inactive lever #1, but exhibited lower lever presses for the lever #2, that is the new active lever, in comparison to GDM. PDM did not attain the same mean level of responsing as represented in grey following reversal. Their sums of active lever presses through FR5 sessions and through rFR5 sessions differed, reflecting also the impaired reallocation of the instrumental response. Data are group means and SEM. [Colour figure can be viewed at wileyonlinelibrary.com].

In order to determine whether the effect of AIC lesions on decision‐making were attributable to deficits in behavioural flexibility or motivation, rats were subsequently trained on an instrumental conditioning task (Fig. 3). All rats quickly learnt to discriminate the active lever from the inactive lever (main effect of lever: *F*_1,28_ = 10 881, *P* < 0.001, *p*η^2^ = 1.00; lever × session: *F*_8,224_ = 2772.0, *P* < 0.001, *p*η^2^ = 0.99), and there were no differences in lever pressing between GDM and PDM or sham and lesioned animals (group: *F*_1,28_ = 1.58, *P* = 0.22; Fig. 3A and C). Following the reversal of contingencies, all rats, successfully reallocated their responding towards the previously inactive, now active, lever (main effect of lever: *F*_1,28_ = 25.69, *P* < 0.001, *p*η^2^ = 0.48 and lever × session interaction: *F*_6,168_ = 242, *P* < 0.001, *p*η^2^ = 0.90; Fig. 3B and D). AIC lesions did not result in any reversal learning deficits as there were no differences in animals’ ability to redirect responses on the active lever (main effect of active lever × surgery group interaction: *F*_1,28_ = 0.4, *P* = 0.53). However, PDM exhibited a reduced number of active lever responses following the reversal, although this only reached significance at a trend level (F_1,28_ = 2.97, *P* = 0.1, *p*η^2^ = 0.1).

**Figure 4:**
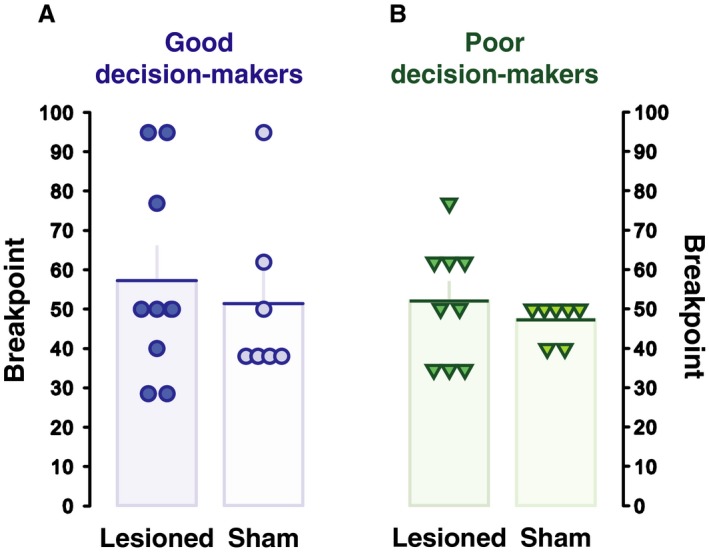
Unaltered general motivation after AIC lesion. (A and B) The breakpoint on a PR schedule did not differ between good and poor decision‐makers and was not influenced by AIC lesions. Data are group means and SEM. Each dot represents an individual. [Colour figure can be viewed at wileyonlinelibrary.com].

The effects of AIC lesions did not produce any overt motivational deficits. When rats were subsequently challenged under a progressive ratio schedule of reinforcement all animals continued to respond on the active lever to a greater extent, but neither decision‐making nor lesion group influenced the motivation for the reinforcer as measured by the breakpoint (main effect of lever: *F*_1,28_ = 57.14, *P* < 0.001, *p*η^2^ = 0.67; lever × group: *F*_1,28_ = 2.16, *P* = 0.15 and lever × surgery interaction: *F*_1,28_ = 1.64, *P* = 0.21; Fig. 4).

## Discussion

Using the rGT, a rodent analogue of the IGT (Bechara *et al*., 2005), we demonstrate that bilateral excitotoxic lesions of the AIC produce bidirectional effects on cost‐benefit decision‐making. Rats that had previously demonstrated the ability to acquire an optimal strategy and predominantly chose from the low risk – low incentive options during the course of a single session were impaired following AIC lesions. In marked contrast, animals that showed a consistent preference for the high risk – high incentive options showed improved performance following AIC lesions.

There are numerous rodent versions of the rGT each with their advantages and relative weaknesses (de Visser *et al*., 2011). Here, we used a task that enabled us to measure the transition from exploration to exploitation in decision‐making over the course of a single session (Rivalan *et al*., 2009). However, this approach does not allow for the expression of stable responding prior to challenges. Therefore, one may argue that the observed effects may simply reflect a regression towards the mean across repeated trials of animals selected in the extremes of the population over a single session. But, as there was no commensurate change in performance over repeated rGT sessions in sham‐treated animals this explanation is unlikely. Similarly, the possibility exists that some animals improved during the second session due to a greater familiarity with the task. However, the task contingencies were altered such that previous simple associative knowledge from the first session could not directly influence performance in the second. Additionally, neither PDM nor GDM groups demonstrated acquisition of an optimal strategy during the exploration phase of the second rGT suggesting these results were not due to learning and lastly, such an explanation could not account for the impairments observed in GDM. As the contingencies were switched in the second, post‐surgery, rGT test, the alterations in performance observed in lesioned rats could have arisen because of a failure to update a response strategy leading to preservative responding, that is GDM may have continued to respond in apertures that were previously associated with advantageous outcomes as they were unable to reallocate responding. However, any potential contribution of a deficit in behavioural flexibility was parametrically controlled in the subsequent instrumental task that revealed that AIC lesions had no effect on the ability of rats to quickly switch response allocation over the course of successive sessions. Similarly, the alterations in performance observed in the rGT following AIC lesions is unlikely to be accounted for by differential motivational effects as there were no differences between groups in, and no effect of lesion on, breakpoint under a progressive ratio schedule of reinforcement (Hodos, 1961).

The present data are consistent with evidence that, in rodents, the insula is involved in the maintenance of the utility of action–outcome associations (Balleine & Dickinson, 1998; Parkes & Balleine, 2013; Parkes *et al*., 2015). In the present study, bilateral lesions of the AIC may have prevented the retrieval of the subjective incentive value of the different options prior to response selection, leading to alterations in responding in animals that had previously expressed strong preferences between risky and safe options. Such an explanation may also be helpful in elucidating the conflicting data from previous studies using other operationalized versions of the IGT in rodents, in which manipulations of the insula were observed to either increase (Mizoguchi *et al*., 2015) or decrease (Pushparaj *et al*., 2015b) risky choice. These studies utilized different behavioural paradigms, but both temporarily silenced the insula, through either pharmacological or chemogenetic methods and importantly, in both examples, either the safe (Pushparaj *et al*., 2015b) or risky (Mizoguchi *et al*., 2015) options were selected relatively infrequently at baseline prior to AIC inactivation. Therefore, in both cases, alterations in AIC function increased choice of an option that was selected relatively infrequently, indicating that AIC inactivation reduced animals state‐dependent bias. In other words, rather than mediating risky choice *per se* on tasks such as the rGT, the insula is instead engaged in parsing subjectively favourable options under conditions of uncertainty or risk. Relatedly, temporary inactivation of the AIC during a rodent slot machine task impairs performance exclusively on trial types when animals’ choice is near‐optimal at baseline (Cocker *et al*., 2016). Furthermore, a recent study has demonstrated that chemogenetic inhibition of the AIC abolishes sensory specific satiety providing further evidence that the AIC facilitates selection of favourable options dependent on the current state of the animal (Parkes *et al*., 2017). Lastly, human subjects that sustain damage to the insula show impairments on a gambling task that are not characterized by a linear increase or decrease in risk preference, but rather a failure to update betting strategies as contingencies change (Clark *et al*., 2008). These data collectively indicate that in both rodents and humans, the AIC is integral in guiding state‐dependent decision‐making.

During the IGT, human subjects tend to initially explore all options and become familiar with the relative contingencies before using that knowledge to maximize point gain during the latter half of the session, the so‐called exploitation phase (Bechara *et al*., 1994). Similarly, in the rGT animals also initially sample from all the ‘decks’ before establishing more restrictive choice strategies. However, animals, like healthy human subjects, vary in the extent to which they demonstrate this shift in strategy away from the larger but costlier ‘decks’ towards the smaller, safer options (Rivalan *et al*., 2013; Steingroever *et al*., 2013). Here, the finding that individual differences in rGT performance influence the effects of AIC lesions suggest that the qualitative, rather than quantitate values of the different options guide behaviour, that is in PDM the valence of the large reward appears to outweigh the larger potential punishment. AIC lesions appear to weaken these subjective choice strategies. For instance, if AIC lesions resulted in quantitative deficits, unidirectional effects would be expected, such as a general increase or decrease in choice of the larger riskier options. However, as the effects were bidirectional, dependent on the previous strategy of the individual, it would indicate a failure to retrieve the qualitative cost/benefit associations that animals used to guide behaviour and consequently choice of either the risky or safe options approached equivalency in both subsets of lesioned animals. This is further demonstrated by the finding that AIC lesions failed to alter behaviour in the intermediate group, who did not display a robust and biased exploitation strategy prior to surgery.

The bidirectional influence of AIC lesions on decision‐making observed here are reminiscent of the bidirectional influence of AIC lesions on the reinforcing properties of cocaine, which has, at least upon initial exposures, both appetitive and aversive properties (Ettenberg *et al*., 1999). We recently demonstrated that AIC lesions prior to drug exposure robustly facilitated loss of control over drug taking. In contrast lesions performed after animals had escalated cocaine intake restored control over drug taking. We suggested that these opposing effects could be due to alterations in the interoceptive states that mediate the aversive properties of cocaine following drug exposure (Rotge *et al*., 2017). This is in line with evidence that pre‐training lesions of the AIC increase seeking responses in a seeking‐taking task for cocaine (Pelloux *et al*., 2013), whereas post‐training inactivations or lesions of different subterritories of the AIC decrease cue‐induced reinstatement of an extinguished instrumental response (Cosme *et al*., 2015; Pushparaj *et al*., 2015a). Overall, these data indicate that the role the AIC plays is fundamentally altered by animals’ previous history with drugs, further emphasizing the state‐dependent nature of AIC recruitment during reward‐related behaviour.

What is not currently clear is whether the AIC contributes to decision‐making (present study), impulsivity and compulsivity (Belin‐Rauscent *et al*., 2016) through independent or inter‐related mechanisms. Likewise, the associated neural circuits that mediate the AIC's influence over these behavioural processes remain unknown. Two candidate circuits involve the amygdala and the nucleus accumbens (NAC), with which the AIC shares extensive reciprocal connections (Augustine, 1996; Reynolds & Zahm, 2005). The AIC‐accumbens circuit has been purported to be a functional interface between emotion states represented by the insula and goal‐directed behaviour organized by the NAC (Mogenson *et al*., 1980). The functional connection between these two regions has been demonstrated to be altered following exposure to addictive drugs (McHugh *et al*., 2013; Seif *et al*., 2013). The AIC‐amygdala circuit appears, at least in humans, to be critically involved in the conscious processing of emotional states related to subjective feelings and impulse control (Craig, 2009a; Xie *et al*., 2011). Further research is warranted to identify the neural circuits by which the AIC exerts influence over decision‐making and impulsive control.

These results indicate a role for the AIC in individual differences in cost‐benefit decision‐making. AIC lesions produced bidirectional effects on rats’ ability to maximize reward depending on animals’ strategies prior to surgery, thereby suggesting that in rats, as in humans, the AIC contributes to guiding behaviour according to internal states. These data may help elucidate the contribution of the insula to inter‐individual differences in interoception and to the vulnerability to psychiatric disorders such as drug addiction.

## Conflict of interest

The authors have no conflict of interest to declare.

## Author contributions

DB, MLD, ABR and JL designed the experiment. ACM wrote the behavioural programs for the rGT. MLD, JL and ABR performed the experiments. MLD, ABR, PJC and DB analysed the data. PJC, MLD, JLH, ACM and DB wrote the MS.

## Data accessibility

Data are available upon request from the corresponding author.
