# Supplementary material for: The anterior insula bidirectionally modulates cost‐benefit decision‐making on a rodent gambling task
Source: Eur J Neurosci. 2017 Oct 6;46(10):2620–8. doi: 10.1111/ejn.13689 (PMC5725664; doi:10.1111/ejn.13689)
Supplement: Supplementary file 1 [file EJN-46-2620-s001.pdf]

EJN European Journal of Neuroscience
FENS Federation of European Neuroscience Societies

The anterior insula bidirectionally modulates cost-benefit decision making on a rodent gambling task

Daniel ML, Cocker PJ, Lacoste J, Mar AC, Houeto JL, Belin-Rauscent A & Belin D

Review timeline:

Submission date:	26 July 2017
Editorial Decision:	14 August 2017
Revision received:	18 August 2017
Editorial Decision:	25 August 2017
Revision received:	27 August 2017
Accepted:	29 August 2017

Editor: Paul Bolam

1st Editorial Decision 14 August 2017

Dear David,

Your manuscript has gone through the 'Rapid Review Process". It was reviewed by two external reviewers as well as by the Editorial team. The reviews collectively indicate that your experiments generated new and important information. However, there are a few issues that need to be clarified/resolved before we can consider your manuscript further for publication in EJN.

As you can see, both reviewers recognised that your study is interesting and performed well. They both raise some minor points that need to be addressed; these are mostly clarifications of the text to make things clearer and are within the category of 'minor revisions'. In addition to these points we also noted the following that need to be addressed to bring it up to 'EJN style'.

- Materials and Methods heading missing.
- Old EU guidelines used.
- We need an explicit statement that institutional ethical approval was granted.
- Precise P values (greater that 0.001) should be reported, even if they are deemed 'not significant'.
- Data statement needed.
- Fig 1 needs to be at a higher resolution.
- Bar charts should be replaced by much more informative scatter plots or similar (as per EJN guidelines).
- Include 'n' in legends to Figs 3 and 4
- Reference list is not in EJN style, please look at recent copies of the journal.

If you are able to respond fully to the points raised, we would be pleased to receive a revision of your paper within 30 days.

Thank you for submitting your work to EJN.

Best wishes,

Paul & John
co-Editors in Chief, EJN

Reviews:

Reviewer: 1 (David Bannerman, University of Oxford, UK)

Comments to the Author
"The anterior insula bidirectionally modulates cost-benefit decision making in a rodent gambling task."
This study investigated the effects of excitotoxic lesions of the anterior insula cortex in rats on a version of

the Iowa Gambling Task (IGT). Lesions had strikingly different effects, depending on pre-operative levels of performance. Animals that had performed well before surgery were impaired by the lesions, whereas animals that had performed poorly originally showed improvements in performance after lesions relative to matched sham controls. There were no effects of the lesions on a progressive ratio task or on either acquisition or reversal of a simple, instrumental lever pressing task.
This is a good study with a very interesting and thought-provoking result. The experiments are conducted appropriately, the data are correctly analysed and the paper is extremely well written. I have very few comments.
My main comment is one of scientific curiosity with regards to the effects of the lesions on the intermediate group (the data for which were discarded and not reported in the manuscript). Was there no effect of the lesion in this group? I appreciate that the inclusion of a third group might make the statistical analysis less clear cut.
Please clarify – were both "advantageous holes " always on the same side? What exactly do the probabilities refer to (is it the probability of reward or the probability of a given delay)?
More detail on the "reshuffle" for the second rat IGT could be provided.

Reviewer: 2 (Francesco Papaleo, Istituto Italiano di Tecnologia, Italy)

Comments to the Author
Overall, this is timely and well-performed set of experiments. I would just clarify a bit better the methods with a schematic figure of the valence of the holes. In particular, it is not clear what is the utility of having 4 nose pokes if the two on the right and the two on the left produced the same effects? If this is not the case, please clarify it. Would not be enough to have just 2 choices then? Moreover, it should be specified if between the 4 holes, the effects were the same within the 2 holes on the right and the two on the left.

There are some typos on the text (e.g. 3rd line of introduction, methods last paragraph etc…)...better to revise all the text.

Maybe authors should highlight that the reversal and motivational control tests were done using the same wall of the reward food magazine and using lever press, which differ from the settings used for the rGT paradigm. Probably this could not be a confounding factor but it is not strictly controlling for parametric settings.

Authors' Response 18 August 2017

We would like to thank you and the reviewers for taking the time to review our manuscript **"The anterior insula bidirectionally modulates cost-benefit decision making on a rodent gambling task".**

We are grateful for the speed of the review and for the constructive comments that address weaknesses of the initial manuscript. In response to the editorial team and the reviewers comments we have clarified aspects of the methods and inserted precise p values. We have also entirely re-formatted the figures as requested.

We feel that the manuscript has been greatly improved by these amendments.

Please find below a copy of the reviewers' comments (in italics) along with our own itemized response.

Editorial team:

The editorial team raised a number of concerns relating to complying with EJN's publication policies, including:

Materials and Methods heading missing. –
Old EU guidelines used. –
We need an explicit statement that institutional ethical approval was granted. –
Precise P values (greater that 0.001) should be reported, even if they are deemed 'not significant'.
Data statement needed. –
Fig 1 needs to be at a higher resolution. –
Bar charts should be replaced by much more informative scatter plots or similar (as per EJN guidelines). –

Include 'n' in legends to Figs 3 and 4 –
Reference list is not in EJN style, please look at recent copies of the journal.

All of these issues have now been rectified accordingly.

Reviewer 1:

Reviewer 1 notes that the study is "interesting" and the paper "well-written" and has the following comments:

My main comment is one of scientific curiosity with regards to the effects of the lesions on the intermediate group (the data for which were discarded and not reported in the manuscript). Was there no effect of the lesion in this group? I appreciate that the inclusion of a third group might make the statistical analysis less clear cut.

The reviewer is correct in their assumption that we did not include the intermediate group in the main analysis as it may confuse the interpretation of the results, particularly as this group is relatively heterogeneous in comparison to the good- and poor-decision making groups. However, we agree that the data from this group is potentially informative and we have thus included an additional analysis of this group in an added sentence (pg 6, pgh 4). These data show that anterior insula lesions produced no behavioural effects on this group further supporting our initial observation that AIC is differentially recruited by rats performing well or very poorly in the task, potentially reflecting inter-individual differences in the reliance on interoceptive control over behaviour.

Please clarify – were both "advantageous holes " always on the same side? What exactly do the probabilities refer to (is it the probability of reward or the probability of a given delay)? More detail on the "reshuffle" for the second rat IGT could be provided.

Both the advantageous options were located on one side of the chamber and the disadvantageous options on the other. Reward is always delivered but may be concomitant with a time-out punishment. We apologise if this was unclear and have now clarified this point in the revised Ms (pg 4-5). On a related note, in subsequent studies we have altered the spatial location of the various options to intermingle both advantageous and disadvantageous options and an exploratory analysis did not reveal any significant differences in performance between cohorts of animals run on the initial vs. the altered task set-up. Thus, having both good options on one side of the chamber, rather than intermixed across the sides, does not appear to make the discrimination any easier to resolve, especially since side bias is controlled for by the extensive exposure to forced choice trials beforehand. Additionally, the strategy implemented in the present study enabled us better to contrast advantageous and disadvantageous options in the re-test sessions when contingencies were reshuffled.

Reviewer 2:

Reviewer 2 mentions that the experiment is "well-performed" and "timely" and has the following concerns:

1, In particular, it is not clear what is the utility of having 4 nose pokes if the two on the right and the two on the left produced the same effects? If this is not the case, please clarify it. Would not be enough to have just 2 choices then? Moreover, it should be specified if between the 4 holes, the effects were the same within the 2 holes on the right and the two on the left.

The use of four holes ensured that animals had to sample from multiple options in order to delineate the optimal strategy. With two holes only the exploration phase of the task, which has been shown (including in this study) to require between 10 and 30% of the total trials, would become a marginal component as the animals would very quickly adopt a bias towards to least punished of the two options. However, as the reviewer notes the overall expected utility of both advantageous/disadvantageous options were equivalent. Which is why in the multiple previous studies using this paradigm they have been grouped together for analysis. As the description of these options came up for both reviewers we have substantially revised that section of the methods in order to make it clearer (pg 4-5).

2, There are some typos on the text (e.g. 3rd line of introduction, methods last paragraph etc…)...better to revise all the text.

The text has been thoroughly proof read and hopefully all errors corrected.

3, Maybe authors should highlight that the reversal and motivational control tests were done using the same wall of the reward food magazine and using lever press, which differ from the settings used for the rGT paradigm. Probably this could not be a confounding factor but it is not strictly controlling for parametric settings.

The rGT was conducted in separate boxes from the reversal learning and motivational paradigms, thus any effects of prior rGT performance were extremely unlikely to have affected performance. We have revised the Ms to make this point more explicit (pg 4, pgh 5).

Once again, we would like to thank the editorial team and reviewers for their insightful and helpful comments. We hope that this improved version of our article is now more suitable for publication in European Journal of Neuroscience.

2nd Editorial Decision 25 August 2017

Dear David,

Your revised manuscript has been re-evaluated by the Editorial team and we are pleased to say that it will be accepted for publication in EJN after you dealt with a couple of minor points. Please give the full name of the ethics committee that granted permission for the work and please provide a graphical abstract.

Thank you for submitting your work to EJN.

Kind wishes,
Paul & John

Paul Bolam & John Foxe
co-Editors in Chief, EJN

Authors' Response 27 August 2017

The full name of the ethics committee has been included in the Methods, and a Graphical Abstract has been provided.
